# Lamin A to Z in normal aging

**DOI:** 10.18632/aging.204342

**Published:** 2022-10-17

**Authors:** Stanley R. Primmer, Chen-Yu Liao, Oona M.P. Kummert, Brian K. Kennedy

**Affiliations:** 1 Independent Affiliation, Lauderhill, FL 33319, USA; 2 The Buck Institute for Research on Aging, Novato, CA 94945, USA; 3 Healthy Longevity Translational Research Programme, Yong Loo Lin School of Medicine, National University of Singapore, Singapore; 4 Centre for Healthy Longevity, National University Health System, Singapore; 5 Departments of Biochemistry and Physiology, Yong Loo Lin School of Medicine, National University of Singapore, Singapore

**Keywords:** lamin A, prelamin A, zmpste24, mTOR, aging

## Abstract

Almost since the discovery that mutations in the LMNA gene, encoding the nuclear structure components lamin A and C, lead to Hutchinson-Gilford progeria syndrome, people have speculated that lamins may have a role in normal aging. The most common HPGS mutation creates a splice variant of lamin A, progerin, which promotes accelerated aging pathology. While some evidence exists that progerin accumulates with normal aging, an increasing body of work indicates that prelamin A, a precursor of lamin A prior to C-terminal proteolytic processing, accumulates with age and may be a driver of normal aging. Prelamin A shares properties with progerin and is also linked to a rare progeroid disease, restrictive dermopathy. Here, we describe mechanisms underlying changes in prelamin A with aging and lay out the case that this unprocessed protein impacts normative aging. This is important since intervention strategies can be developed to modify this pathway as a means to extend healthspan and lifespan.

## INTRODUCTION

The *LMNA* locus is complex, with variations in polyadenylation and splicing leading to the generation of all A-type lamins, which are linked to myriad nuclear structural roles and functional properties [1–3]. B-type lamins are encoded by other loci and have been attributed overlapping functions. A-type lamins have received the bulk of the attention, however, since mutations at this locus have been linked to a variety of dystrophic and progeroid syndromes [1–3]. The purpose of this review is not to provide a thorough overview of A-type lamin functions and their disease connections, but rather to evaluate one hypothesis: that altered processing of lamin A due to a reduction in Zmpste24 (aka FACE1) with age promotes aspects of the normal aging process. Several reviews have been cited herein for readers interested in broader questions around A-type lamin function and dysfunction.

Transcription of the *LMNA* gene produces mRNAs primarily for two proteins: prelamin A and lamin C. Prelamin A undergoes 4 post-translational steps to create mature lamin A for which the endoprotease, Zmpste24, is essential [4–7]. The prelamin A protein contains a CAAX (Cysteine-Alipathic-Alipathic-any amino acid) box at its C-terminal end (see [8, 9] for review). In humans and mice, the CAAX box of prelamin A is CSIM. The first step is farnesylation of cysteine, which is followed by cleavage of the terminal 3 amino acids by Zmpste24 or Rce1. The third step is carboxymethylation of the same cysteine. Zmpste24 is then the only known protease that can delete an additional 15 amino acids to form mature lamin A protein. If there is an imbalance with insufficient Zmpste24 to process all of the prelamin A, prelamin A accumulates in the nucleus [10].

The mutation most associated with Hutchinson-Gilford progeria syndrome is a non-coding single amino acid substitution that activates a rarely used splice site in the C-terminus of the protein. This interferes with C-terminal cleavage of lamin A by Zmpste24 resulting in a permanently farnesylated lamin A, termed progerin, which acts in a dominant fashion to promote a range of accelerated aging phenotypes comprising Hutchinson Gilford progeria syndrome (HGPS) [2, 3]. Much debate has centered on whether progerin, which is generated at low levels in absence of HGPS mutations, contributes to normal aging [11–14]. Progerin expression has been observed at very low levels in normal cells and may accumulate with age, although the latter assertion has been hard to verify [15–17].

The toxic effects of nuclear prelamin A are similar to that of progerin and resemble aspects of premature aging, but with a slower onset than progerin [18, 19]. Here, we present evidence supporting the hypothesis that declining levels of Zmpste24 with age contribute to an elevated level of prelamin A and it may be this lamin A variant that drives aspects of normal aging pathology.

## Zmpste24 and accelerated aging

In both human patients and animal models, mutations leading to the expression of progerin cause a segmental progeria syndrome in which a subset of features of accelerated aging are present [11, 20]. This is also the case for mice lacking *ZMPSTE24^−/−^*, which survive about 5 months of age [6] and show both molecular and physiologic features of accelerated aging. These include genome instability [21], age-related bone loss [18], oxidative damage [22], cell senescence [19], altered epigenetic patterns [23], similarities in skeletal muscle decline [24], reduced adult stem cell function [25, 26], and altered age-related cell signaling pathways [27, 28]. Many of these phenotypes are described in more detail below (Table 1). Patients with the laminopathy, restrictive dermopathy (RD), have mutations in either *ZMPSTE24* or *LMNA*, the latter associated with altered processing and the accumulation of prelamin A [4, 29]. RD has some phenotypes of accelerated aging; however, the condition is often very early onset and severe, making comparison with normal aging more challenging.

**Table 1:** Phenotypes associated with prelamin a expression that are associated with aging and/or progerin expression.

**Phenotype associated with prelamin a expression**	**Aging**	**Progerin expression**	**Reference(s)**
Autophagy Defects	✔	✔	[27, 125]
Cellular Senescence	✔	✔	[10, 125–127]
DNA Damage	✔	✔	[21, 67, 125, 128]
Dysmorphic Nuclei		✔	[10, 126, 129, 130]
Epigenetic Dysregulation	✔	✔	[131]
Heterochromatin Alterations	✔	✔	[30, 56, 110]

## Evidence for reduced expression of *zmpste24* during aging – *in vitro* studies

**Figure 1:**
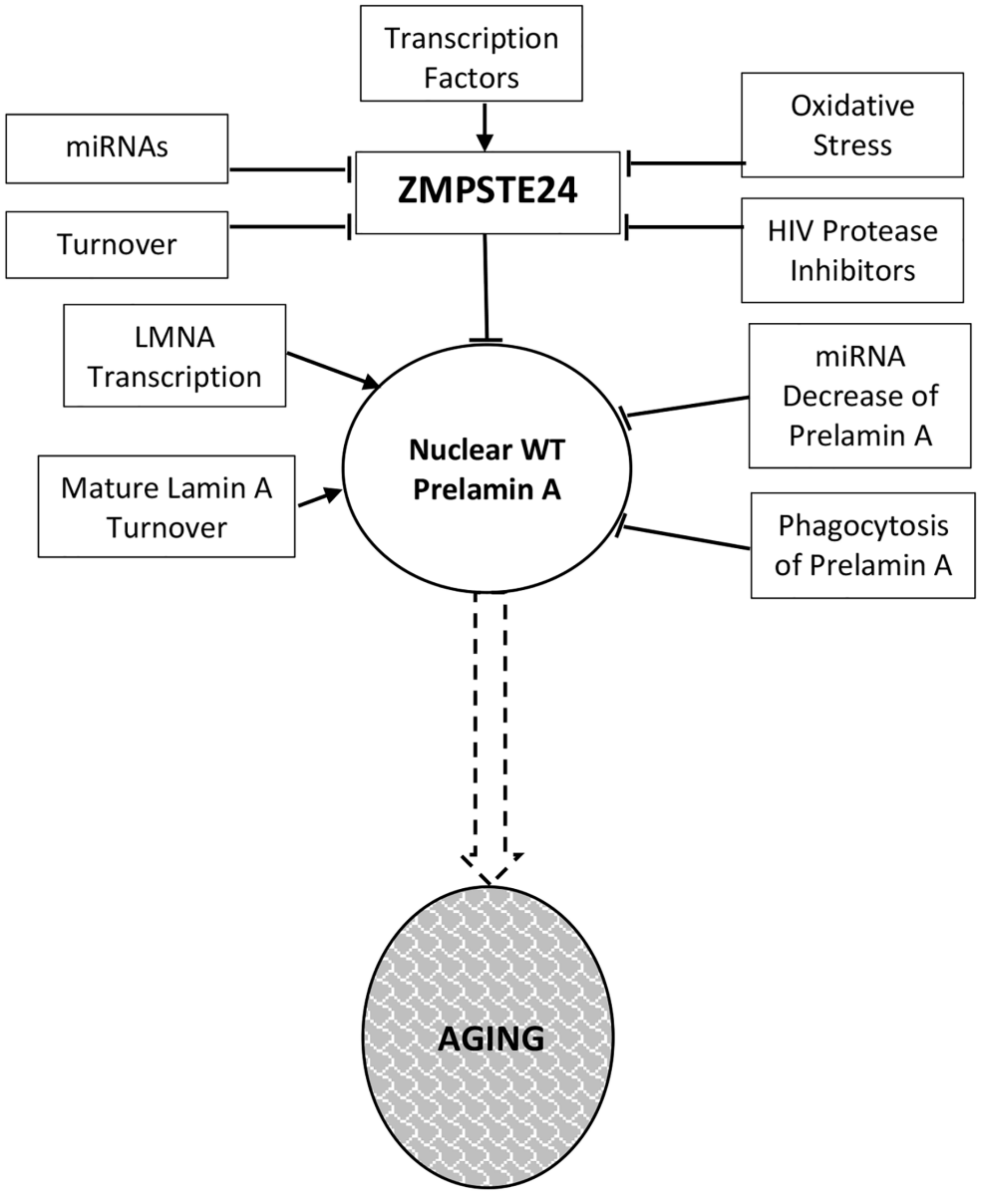
**Prelamin a regulation in normal aging.** Levels and activity of Zmpste24 are regulated in several ways related to aging. Reduced enzymatic activity is proposed to lead to increased levels of prelamin A and consequent aging phenotypes. These pathways are described throughout the text.

Much of the current data supporting a decline in Zmpste24 protein levels during the aging process comes from analysis of cells *ex vivo*. Here, we summarize that data while emphasizing the need for *in vivo* studies in mice and human tissue samples. Figure 1 details the regulatory events that dictate Zmpste24 levels and activity during aging.

### Skin fibroblasts of centenarians

A study of skin fibroblasts collected from young (8–35) and older (65–80) individuals, as well as centenarians (95–105) was performed and, after 6 passages in culture, the cells were assayed for aging properties [30]. Evident in cells from centenarians (over 100), but not 65–80 year old individuals, is reduced mRNA and protein levels of Zmpste24 and an accompanying increase in unprocessed lamin A. One challenge of studying centenarians is that the control group, people from the same generation who did not age well, are not around from which to collect material. Thus, when changes are observed such as those described above, two potential explanations are possible. First, it may be that *ZMPSTE24* expression declines with age, implying that this is a property of normal aging. Since levels are not reduced in the 65–80 year old group, this would presumably be a late event in aging. Second, it is possible that centenarians have low levels throughout life and that this serves some protective role. We favor the former, given that other evidence indicates that Zmpste24 levels decline with age, as discussed below, but clearly more studies need to be performed.

### Fetal lung fibroblasts

Human primary fibroblasts derived from fetal lung are often used for cell senescence studies. These cells will undergo senescence after prolonged serial passaging, or in response to a range of other induction methods. The level to which *in vitro* replicative senescence resembles aspects of normal physiologic aging remains debated [31]; however, recent studies indicate that *in vivo* cell senescence is a significant driver of aging in large part because of their unique secretory profiles termed the Senescence Associated Secretory Profile (SASP) [32]. One report has indicated that *ZMPSTE24 (FACE-1)* mRNA levels decline during cell senescence induced through serial passaging in these cells [33]. This finding is intriguing and although correlative, the decrease of Zmpste24 protein and resulting increase in prelamin A indicate cause and effect, but studies should be repeated in other primary fibroblast isolates and also when senescence is induced through other methods.

### Mesenchymal stem cells

Mesenchymal stem cells (MSCs), which can be isolated from bone marrow, adipose and other tissues, are multi-potent, giving rise to cells in a range of tissues. These include bone, adipose, smooth muscle, cardiomyocytes, and other tissues that overlap significantly with those affected in laminopathies, making them interesting cells to study in this context. In addition, expression of progerin alters the differentiation properties of MSCs *in vitro*, impairing adipose differentiation. This is intriguing given that loss of adipose tissue is a hallmark of many laminopathies [34–36]. Also intriguing is that overexpression of wild-type lamin A conferred similar phenotypes to that of progerin, although to a lesser extent. One potential reason for this is that in the context of overexpression, the amount of lamin A produced may outpace the ability of Zmpste24 to confer processing, leading to elevated prelamin A. However, it cannot be ruled out that excess mature lamin A may disrupt nuclear functions through other mechanisms.

Enforced overexpression of prelamin A in human MSCs has also been reported to lead to elevated levels of proteins associated with osteogenesis [37]. This is surprising since *ZMPSTE24^−/−^* mice have higher levels of adipogenesis [18, 38] and osteoporosis [39]. Moreover, cells in aging bone marrow are thought to skew toward adipogenesis and away from osteogenesis [40, 41]. One clue to explain this apparent discrepancy may come from observations that older wild-type mice were found to have low levels of mature lamin A/C in osteoblasts [38], which could be an indirect consequence of loss of *ZMPSTE24*, at least with regard to mature lamin A. Knockdown of Lamin A/C was associated with increased adipogenesis [38]. Low levels of mature lamin A may counteract the effects of increased osteogenic factors observed in prelamin A overexpression. The MSCs are not immortal in culture and undergo replicative senescence. As with fibroblasts, senescence in these cells is associated with down-regulation of *ZMPSTE24* and nuclear accumulation of prelamin A [42]. The mechanism involves upregulation of the microRNA miR-141-3p, which targets the 3′ UTR of *ZMPSTE24*, leading to reduced expression. Enforced expression of miR-141-3p induced senescence in cultured MSCs and injection of the microRNA in mice led to decreased liver expression of *ZMPSTE24*. The activity of HDAC1 and HDAC2 declines during replicative senescence and decreases the expression of *ZMPSTE24* by upregulating miR-141-3p [42]. Increased prelamin A expression has also been proposed as a senescence marker to screen MSCs *in vitro* before clinical application [43]. These findings reinforce the studies in fibroblasts that reduced expression of ZMPSTE24 is associated with cell senescence and call for a wider analysis of the microRNA in tissues from aging animals.

A more recent paper found reduced Zmpste24 levels and prelamin A accumulation during cell senescence in subchondral bone mesenchymal stem cells [44]. Enforced expression of prelamin A in these cells accelerated senescence, which was associated with DNA damage, including at telomeres, and increased expression of inflammatory factors. Interestingly, the accelerated senescence phenotype could be suppressed by vitamin C, which also reduced inflammatory factors.

### Endothelial cells

Premature senescence in primary human epithelial cells isolated from human umbilical vein or cord blood, can also be induced by elevated levels of prelamin A, this time induced by exposure of cells to protease inhibitors that inhibit activity of Zmpste24 [45], which evokes a similar phenotype in human bone marrow-derived MSCs [46]. Interestingly, this phenomenon was observed in both precursor and mature endothelial cells.

### Vascular smooth muscle cells

The Shanahan lab found that nuclear accumulation of prelamin A in VSMC of arterial media with age and *in vitro* was correlated with the down regulation of *ZMPSTE24* (See Figure 1 in [47]). These effects occurred both *in vitro* and in cells from old individuals processed *ex vivo*. The decline of ZMPSTE24 and the increase in nuclear prelamin A occurred before cellular senescence. The presenescent phase in VSMC included modification of migrational characteristics [48] and calcification due to increased levels of the osteogenic markers Runx2, ALP, and osteocalcin resulting primarily from greater DNA damage [49]. SASP factors were secreted by VSMC with higher levels of prelamin A. This finding is consistent with an early study that protease inhibitors that impair Zmpste24 function lead to premature senescence in VSMC [50].

The importance of the role of VSMC in atherosclerosis is increased by the report that VSMC may transdifferentiate into macrophage-like cells when proliferating into plaque in the intima in response to arterial damage or stress [51]. VSMC contribute with macrophages to foam cells in lesions in arteries [51, 52]. Prelamin A may have a role in these events. When endothelial cells from human umbilical vein or cord blood were treated with the HIV protease inhibitor Atazanavir, which inhibits Zmpste24, the authors observed nuclear accumulation of prelamin A leading to irregularly shaped nuclei, premature cellular senescence, and an increase in monocyte adhesion. The complexity of atherosclerosis involves many factors in addition to prelamin A in VSMC, foam cells, and endothelial cells as described in many articles [45, 53, 54]. The review of the role of lamins in atherosclerosis by Jiang and Ji provides additional useful information [55].

## *In vivo* studies

### Pilot studies

**Figure 2:**
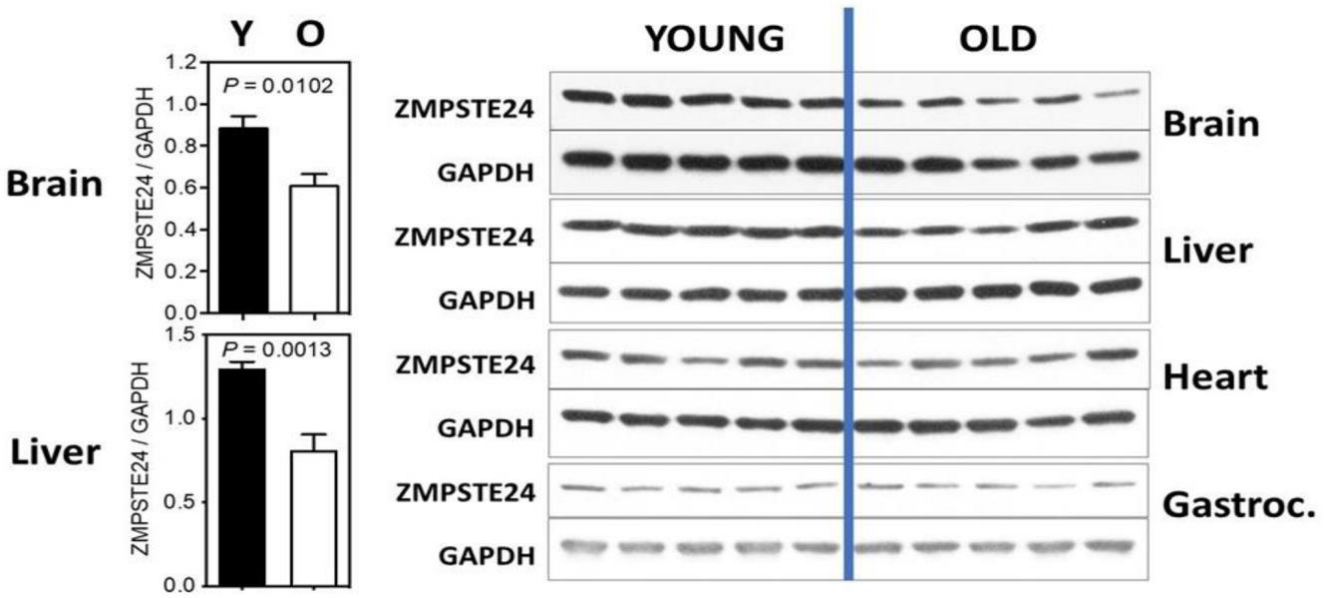
**ZMPSTE24 protein expression during murine aging.** ZMPSTE24 protein levels were determined by Western blots from brain, liver, heart, gastrocnemius, and subcutaneous fat of young (9 months of age, *n* = 5) and old (22 months of age, *n* = 5) female mice. Relative ZMPSTE24 levels (normalized to GAPDH) were quantified by densitometry using ImageJ software (http://rsb.info.nih.gov/ij/). All bars represent mean ± SEM. The statistical significance of differences between two groups (as indicated with *P* values) was determined using unpaired, two-tailed Student’s *t-*test. The antibody used was a Rabbit polyclonal to ZMPSTE24 from Abcam; Catalog number ab38450.

We have recently examined the levels of Zmpste24 in several tissues comparing mice of 9 and 22 months of age. While not definitive at this point, the studies strongly suggest age-associated changes in a tissue-dependent manner and call for further studies to be performed. We have included them to support the overall hypothesis and stimulate further research. Four tissues were examined: brain, liver, heart and gastrocnemius (skeletal muscle) and results are shown in Figure 2. In both brain and liver, we detect a significant decline in Zmpste24 levels, although this was not detected in heart and skeletal muscle. This finding calls for studies across a wider age range and in more tissues, and the levels of Zmpste24 should be correlated with prelamin A.

## Aging mechanisms

### Cellular senescence

Studies above indicate that declining Zmpste24 levels lead to senescent pathology in a variety of cellular contexts, raising the question of how this occurs at the mechanistic level. Several models have been proposed. Classical pathways associated with senescence involve the p53 and pRB-p16^INK4A^ pathways. Activation of p53 and expression of p16^INK4A^ lead to cell cycle arrest and senescence, and ablation of these pathways allow cells to escape senescence and become immortalized. p16^INK4A^ is induced in multiple contexts associated with enhanced prelamin A expression [42, 47, 49, 56, 57], as is induction of p53 [19, 58]. Notably, induction of p16^INK4A^ is associated with all methods to induce senescence in cell culture and, therefore, it is not surprising in the prelamin A context. This raises the question of what is happening upstream of induction of these factors.

Casein kinase 2 may be a factor linking prelamin A accumulation to cellular senescence. CK2 has long been known to be nuclear matrix-associated [59], but a recent paper shows more direct connections with lamin A, which binds to CK2 and inhibits its activity. Loss of lamin A expression leads to enhanced CK2 activity, where prelamin A accumulation is associated with its inhibition [60], in turn leading to senescence induction [60–62]. Down-regulation of CK2 is also associated with accelerated aging and oxidative stress in *C. elegans* [63]. Interestingly, the pro-longevity compound spermidine, which has been reported to activate CK2, was found to suppress cellular senescence in *ZMPSTE24^−/−^* MEFs and to extend the lifespan of the mice from which the cells were derived [60].

Another pathway linking prelamin A to senescence involves p62, a component of the autophagic machinery that has been linked to the aging process [64]. Enhanced autophagy, a process that involves the clearance of damaged cellular macromolecules and structures, has been implicated as a mechanism by which progerin, and more recently prelamin A, is degraded by mTOR inhibition (see below). In the context of exogenous progerin expression or reduced ZMPSTE24 expression in MSCs, DNA damage permits GATA4 to avoid p62 binding and selective degradation [65, 66]. Stabilization of the transcription factor GATA4 leads to monocyte chemoattractant protein-1 (MCP-1 expression), induction of the senescence-associated secretory phenotype and paracrine senescence [65].

### DNA damage

One major candidate mechanism for the manner by which prelamin A promotes cellular senescence involves the induction of DNA damage. DNA damage, in multiple forms, or the induction of the DNA damage response have long been reported to induce cellular senescence. Several reports link expression of prelamin A to DNA damage [67], and some mechanistic studies have been conducted. For instance, cells from mice lacking *ZMPSTE24* were reported early on to have elevated levels of DNA damage [21]. This phenotype has also been observed in fibroblasts from Restrictive Dermopathy patients, which have homozygous mutations in the enzyme [68]. In particular, the latter study was able to identify an increase in DNA double strand breaks [68], and both studies found defective DNA damage responses. A later study also reported increased DNA damage in smooth muscle cells expressing prelamin A [47]. Follow up studies in these cells point to a possible mechanism whereby lamin A/C is part of the DNA damage response and accumulation of prelamin A interferes with the normal role of the intermediate filament proteins [69].

DNA replication stress, in some respects a specialized form of replication stress, is not often considered. However, studies of replicative lifespan in yeast have indicated that a major driver of aging is DNA replication stress [70, 71]. Nuclear lamins have long been suspected to facilitate DNA replication [72], and recent studies have indicated that progerin or prelamin A can induce replication fork stalling, which leads to DNA breaks [73–75]. Interestingly, this leads to activation of the cGAS/STING cytosolic DNA sensing pathway and an interferon response [76]. This is perhaps consistent with increased inflammation associated with loss of *ZMPSTE24* or expression of progerin, which has been previously reported [77]. Treatment of progerin expressing cells with calcitrol, an active version of vitamin D, reduces replication stress and the associated innate inflammatory response [78]. cGAS/STING signaling has been recently linked to cellular senescence and aging [79, 80], making this a pathway to explore in more detail in progeria and normal aging.

### Oxidative stress

Links between oxidative stress and aging date back to the famous hypothesis by Denham Harman [81], although whether oxygen free radicals drive aging remains a matter of debate. While free radicals drive damage to a variety of cellular molecules, they also mediate critical signaling pathways, making it difficult to interpret their net effect on the aging process. Oxidative stress is also linked to a range of age-related diseases and can induce cellular senescence [82].

Increased oxidative stress is linked to reduced Zmpste24 activity [57, 83, 84], and overexpression of prelamin A in mesenchymal stem cells [85]. Interestingly, oxidative stress leads to reduced levels of Zmpste24 [47, 84, 86], possibly through upregulation of miR-141 [42]. This creates a possible feed-forward loop with oxidative damage that accumulates during aging leading to a reduction in Zmpste24 activity, which, in turn, results in more oxidative damage. More studies need to be performed in physiologic oxygen of 2 to 5% rather than in atmospheric oxygen conditions, in order to more closely resemble the *in vivo* environment.

### Sirtuins

A-type lamins have been reported to interact with multiple Sirtuins, protein deacetylases linked to control of healthspan and lifespan [87]. With regard to SIRT1, lamin A binding leads to SIRT1 activation; however, the interaction is reduced in the presence of prelamin A, contributing to adult stem cell decline in *ZMPSTE24^−/−^* mice [88]. Lamin A also binds to and activates SIRT6, which leads to enhanced DNA repair. Again, this interaction is compromised at least in the presence of progerin (prelamin A was not reported). This may be particularly relevant as SIRT6 deficiency leads to a progeroid phenotype and overexpression of the deacetylase leads to lifespan extension [89, 90]. In culture, HGPS fibroblasts have reduced SIRT6 levels and restoration of its expression led to reduced senescence phenotypes [91]. Interestingly, SIRT6 is known to suppress LINE1 retrotransposon activation [92], which has been shown to mediate progression of cellular senescence both in *SIRT6^−/−^* and aging wild-type mice in a manner involving cGAS/STING signaling (see DNA damage section) [93, 94]. SIRT7 also represses LINE1 elements and interacts with lamin A, although whether prelamin A shows altered binding has not been reported [95].

### Autophagy and mTOR signaling

The mTOR signaling pathway, of which regulation of autophagy is one major downstream pathway, is highly linked to aging [96, 97]. mTOR is a nutrient-responsive kinase that evidence indicates is aberrantly upregulated during aging. Reduced mTOR signaling, mediated genetically or with the highly specific drug rapamycin, extends lifespan in a wide range of model organisms. Evidence suggests that mTOR inhibition can also reverse aspects of aging in human studies [98, 99].

These findings make it obvious that mTOR signaling would be examined in progeria models. Initial studies in fibroblasts expressing progerin indicate that rapamycin can enhance autophagy, which is beneficial at least in part because it facilitates clearance of progerin itself [100, 101]. It was later shown to enhance cellular proliferation and reduce levels of cell senescence [102]. These studies have primarily focused on progerin, but a recent study indicates that expression of prelamin A confers similar phenotypes, and adds to previous observations by showing that the protein stimulates mTOR activation and impairs autophagy [27]. These findings suggest that more studies are needed to understand the role of mTOR in progeria and prelamin A-related normal aging.

### Nucleoplasmic reticulum

When there is insufficient Zmpste24 to process prelamin A to the mature form, the nucleus may become dysmorphic [68, 103], display evaginations [104], or contain nucleoplasmic reticulum [105–107]. The term nucleoplasmic reticulum is applied to long, tubular channels that extend deep into the nucleoplasm or even pass entirely through the nucleus [107]. Some are short stubs while others are complex, branching structures. Some terminate at or near nucleoli. The nucleoplasmic reticulum is of particular interest here because it is formed during interphase by excess nuclear prelamin A, as demonstrated when Interphase prelamin A was experimentally produced by suppressing *ZMPSTE24*, either by siRNA or by application of an HIV protease inhibitor (PI) such as saquinavir [105, 106]. When saquinavir was removed from the media, processing resulted in mature lamin A and the number of nucleoplasmic reticulum invaginations was markedly reduced [106]. The role of nucleoplasmic reticulum formation in aging remains poorly understood and more studies are needed in aging organisms.

Stepping back, there are also numerous studies linking progerin, and to a lesser extent prelamin A, expression to loss of heterochromatin. Links to progerin are thoroughly described in a recent review [108]. Regarding prelamin A, early studies linked prelamin A to loss of chromatin organization [109, 110]. More recently, studies of lamina-associated domains (LADs) in *Zmpste24^−/−^* mice indicate altered associations with transcription factors, including Foxa2 that are similar to those of old wild-type mice [111]. More studies are needed, but understanding the heterochromatin alterations associated with altered lamin A function remains a vital area of research.

### Other functions of zmpste24

Given that loss-of-function mutations in *ZMPSTE24* give rise to phenotypes resembling those associated with *LMNA* mutations, many have assumed that the role of Zmpste24 is restricted to modifying processing of Lamin A; yet this may be too simple as other functions of Zmpste24 are known. For instance, other substrates of Zmpste24 have been identified, including proteins that are not prenylated, although the significance of these events remain largely unknown [112]. Notably, Zmpste24 assists in helping translocon pores in the endoplasmic reticulum function smoothly [113, 114]. Translocons can become clogged when proteins enter but fail to properly transverse the pore. Under these conditions, Zmpste24 cleaves clogged proteins into peptide fragments for clearance. This function may have roles in aging, where protein misfolding is increased. A recent study, however, found that *ZMPSTE24* disease mutations all affected lamin A processing but only some mutants interfered with the ability of the enzyme to clear clogged proteins from the translocon [115].

Independently of its enzymatic functions, Zmpste24 also interacts with the interferon-induced transmembrane protein (IFITM) family and facilitates the role of these proteins in blocking entry of enveloped RNA and DNA viruses [116, 117]. Zmpste24 appears to impair entry of a range of viruses, including influenza A, Zika and COVID-19 [118, 119]. As a result, mice lacking *ZMPSTE24* have increased viral loads and show sensitivity to influenza infection. Whether reduced Zmpste24 levels with age contribute to the increased sensitively of older individuals to viruses remains to be determined.

### Questions to be addressed

We propose the following: (1) A-type nuclear lamins are involved in normal aging as well as progeria and (2) it is prelamin A resulting from declining levels of Zmpste24 that drives aspects of normal aging more than expression of progerin. We have described a variety of supporting evidence; however, comprehensive studies remain to be performed to validate this approach. It is critical to address this question given the dramatic increase in the aging population worldwide and the accompanying chronic diseases for which aging is the biggest risk factor. If the lamin A processing pathway can be validated as a driver of normal aging, a variety of new therapeutic approaches will be feasible to extend healthspan. Moreover, this may explain the longevity benefits associated with known interventions, including mTOR inhibitors and Sirtuin activators, among others.

To validate this theory, more comprehensive studies are needed to confirm that Zmpste24 levels and activity decline with aging in animal models and humans and that this is associated with elevated prelamin A levels. These studies need to be performed under optimal conditions (for instance physiological oxygen levels for cell culture) and with the best possible reagents without which interpretation of results is more complicated. Antibodies specific for prelamin A include 3C8 [120] and PL-1C7 [9]. Antibodies specific for Zmpste24 include PA1-16965 [113], 205-8C10 (Daiichi Chemical), and ab38450 (Abcam). We also note that many publications show prelamin A levels without accompanying levels of lamin A and/or without detection of a slower migrating prelamin A band. This precludes determination of ratios of different lamin A isoforms. It may be the ratio of prelamin A to mature A-type lamins that best define associated phenotypes and this should be measured in research studies, if at all possible.

In mice, Zmpste24 and prelamin A levels should be carefully examined in multiple tissues of mice at a variety of ages. Ideally, a variety of associated factors should be analyzed, including levels of (1) other lamin isoforms, (2) microRNAs associated with A-type lamin and *ZMPSTE24* expression, (3) senescence factors and (4) markers of the activity of related pathways such as mTOR. If, as expected, declines in Zmpste24 levels are observed, it may be worth engineering mice lacking the 3′UTR sites for miR-141-3p and miR-335 binding in *ZMPSTE24* [42]. Another genetically modified mouse model of interest would be engineered to overexpress *ZMPSTE24* systemically or in a tissue-specific manner. The prediction would be that these mice would have improved healthspan and lifespan.

Human studies are also needed, including further analysis of cells isolated from humans at different age ranges for the same parameters as those described for murine studies. In addition, muscle or skin biopsies should be tested from different age ranges. Muscle may be of particular importance since mTOR signaling is known to increase in this tissue with age [121]. Finally, human longevity intervention studies should consider examining the levels of prelamin A and Zmpste24 whenever possible. Interventions could include microRNA therapeutics, for instance mimicking the effects of *miR-9* to reduce LMNA expression [122, 123] or inhibiting miR-141-3p through antimiRs [42, 124], strategies to enhance degradation of prelamin A, for instance by driving autophagic clearance [101], or altering transcription levels of *ZMPSTE24*. The appropriate strategy will clearly await a better mechanistic understanding regarding the reasons Zmpste24 levels decline with age.

Understanding the pathways that modulate the aging process is critical toward developing strategies to extend human healthspan, and to assist individuals with progeroid disorders. It has long been debated to what extent the mechanisms of aging and progeria overlap. Loss of Zmpste24 may be a connecting feature and, if correct, serve as a target for present and future longevity interventions.
